# Patients' Self-Disclosure Positively Influences the Establishment of Patients' Trust in Physicians: An Empirical Study of Computer-Mediated Communication in an Online Health Community

**DOI:** 10.3389/fpubh.2022.823692

**Published:** 2022-01-25

**Authors:** Jusheng Liu, Jianjia He, Shengxue He, Chaoran Li, Changrui Yu, Qiang Li

**Affiliations:** ^1^School of Information Management and Engineering, Shanghai University of Finance and Economics, Shanghai, China; ^2^Business School, University of Shanghai for Science and Technology, Shanghai, China; ^3^Center for Supernetworks Research, Shanghai, China; ^4^Shanghai Institute of Public Diplomacy, Shanghai, China; ^5^School of Economics and Management, Shanghai University of Sport, Shanghai, China

**Keywords:** e-health, self-disclosure, social support, physician-patient trust, media richness, computer-mediated communication

## Abstract

With the development of telemedicine and e-health, usage of online health communities has grown, with such communities now representing convenient sources of information for patients who have geographical and temporal constraints regarding visiting physical health-care institutions. Many previous studies have examined patient–provider communication and health-care service delivery in online health communities; however, there is a dearth of research exploring the relationship between patients' level of self-disclosure and the establishment of patients' trust in physicians. Consequently, this study aims to explore how patients' self-disclosure affects the establishment of patients' trust in physicians. “Good Doctor,” which is a China-based online health community, was used as a data source, and a computer program was developed to download data for patient–physician communication on this community. Then, data for communications between 1,537 physicians and 63,141 patients were obtained. Ultimately, an empirical model was built to test our hypotheses. The results showed that patients' self-disclosure positively influences their establishment of trust in physicians. Further, physicians' provision of social support to patients showed a complete mediating effect on the relationship between patients' self-disclosure and patients' establishment of trust in physicians. Finally, evidence of “hope-for-help” motivation in patients' messages weakened the effect of patients' self-disclosure when physicians' social support was text-based, but strengthened it when physicians' social support was voice-based.

## Introduction

With the continuing development of health information technology (1) and telemedicine, online health communities (OHCs), such as Good Doctor (haodf.com) and Chunyu Doctor (chunyuyisheng.com), are becoming increasingly popular online platforms through which patients and physicians can communicate and exchange information (2–6). OHCs provide patients with an online health consultation service, which allows them to communicate with physicians in order to obtain medical information and services; meanwhile, OHCs can provide physicians with economic and social returns (e.g., such as reputation, if the physicians service the patients on OHCs, the physicians can obtain external reputation which is distinct from offline service, such as the electronic votes, gifts, thanks letters from patients) (7). OHCs are extremely convenient for patients who have geographical and temporal constraints regarding physically contacting health-care professionals (8). Overall, the widespread use of OHC facilitates physician–patient communication and improves the accessibility of health-care services.

When compared with offline, face-to-face communication, OHCs represent extremely convenient resources through which patients can investigate diseases and symptoms. However, as OHCs are internet-based, all contact is virtual; thus, OHCs do not afford tangible diagnoses, such as inspections involving physical appraisals (e.g., palpation) or examinations of symptoms that can only be heard or smelled. Therefore, on OHCs, patients' level of self-disclosure is essential for helping the physicians make appropriate medical decisions. However, dialogue on OHCs occurs through computer-mediated communication (CMC), and combining CMC with non-tangible (i.e., non-face-to-face) diagnoses may lead to patients having less trust in physicians when compared to face-to-face communication and offline diagnoses. Thereby, on OHCs, how to improve the patient-physician trust based on the patients' self-disclosure is important to the patients and the physicians.

Self-disclosure refers to information one person communicates to another and, in the social-psychology context, is considered a kind of social behaviour (9). Previous studies have found that, when compared with face-to-face communication, users of CMC tend to disclose more personal information, mainly as a result of the anonymity such services afford (10–12). Thus, along with being distinct from face-to-face communication and representing a novel and unique method of performing self-disclosure, CMC can, in certain contexts, increase the degree self-disclosure, which could contribute to building trust (13). Therefore, it is essential to explore the relationship between self-disclosure and trust in patient–physician CMC in the OHC context. As to patient–physician trust, Gabay (14) found that perceived participative communication (PPC) can improve patient-physician trust, and the patients' perceived control over health positively moderates the relationship between PPC and patient-provider trust. Meanwhile, the physicians' good listening abilities and impartial concern for patients' well being are important factors that can increase the patients' trust in physician (15). Furthermore, Gabay (16) also discussed the communication barriers to trust (e.g., underrating patient's autonomy and lack of attentive listening) and proposed a way of patient-centred communication to improve patient's self-worth and trust. These studies provide well enlightenment for us to understand patient-physician trust. In real life, when a patient consults a physician through an OHC, the patient's trust in the physician develops gradually during the subsequent slow communication process (17). Notably, a previous study has shown that longer information exchange through CMC is more likely to foster trust (18). Therefore, after multiple rounds of CMC-mediated interaction, the physician and patient should develop an understanding of each other and a trusting relationship. From the patient perspective, trust in the physician is influenced by how much information they disclose to the physician (19, 20). Therefore, high patient self-disclosure is important for the establishment of physician–patient trust. Moreover, social support, which refers to interpersonal relations and, in this context, usually concerns emotional and informational support, is another important element in the OHC context, and can affect cooperation and relationships between physicians and patients. Receiving social support from physicians not only benefits patients' mental health (21), but can also, the field of e-health, play a significant role in patients' self-disclosure and trust (22).

Multiple studies across several fields have directly explored self-disclosure, social support, and trust (23–25); however, there is a dearth of research exploring the mechanisms that influence self-disclosure, social support, and trust. Moreover, little research has explored the mediating role of social support from physicians in the relationship between patients' self-disclosure and their building of trust in physicians. In the present research, we explore the effect of patients' self-disclosure on their trust in physicians, as well as the mediating role of social support from physicians in this relationship. Additionally, showing hope of receiving help when asking questions of physicians can not only indicate to physicians the type of information the patients desire, but may also help patients have good CMC experiences (26). For example, questions such as *Do I need an operation?* and *How should I take the medicine?*, which indicate a hope for help, may help physicians accurately and quickly understand the patients' requests, and also help the physicians provide better social support to the patients. However, existing research on the role of patients' “hope-for-help” motivation in patient–provider communication is sparse. Therefore, this study also investigates the moderating role of patients' hope-for-help motivation on the relationship between patients' self-disclosure and physicians' social support in patient–provider communication.

To explore how to build, and the factors that affect, patients' trust in physicians during online patient–provider communication, this research explores the following three questions:

RQ1: *How does patients' self-disclosure affect the establishment of patients' trust in physicians?*
RQ2: *What is the role of social support from physicians in the relationship between patients' self-disclosure and the establishment of patients' trust in physicians?*
RQ3: *What is the role of patients' hope-for-help motivation in the relationship between patients' self-disclosure and physicians' provision of social support?*

To answer the above questions, this study constructed a moderated mediation model to verify the relationship between patients' self-disclosure and the establishment of patients' trust in physicians. To the best of our knowledge, this is the first study to explore patients' self-disclosure and trust during CMC on OHCs. The research findings may provide some insights into physician–patient trust and patient–provider communication in telemedicine and e-health.

## Theoretical Background And Hypotheses

### Physician–Patient Computer-Mediated Communication in Online Health Communities

CMC originated from the “computer supported cooperation” in the 1980s. With the progress of electronic communication technology and the increase of the human being's cooperation, to communicate conveniently, more and more people begin to use the emerging communication tools to communicate and carry out cooperation based on computers. Later, this communication is called computer-mediated communication, which is abbreviated as CMC. At present, CMC is widely regarded as a new communication mode for people to search, transmit, process and communicate with each other under the help of the Internet. There are three basic communication elements in CMC: the disseminator, the media, the receiver of information. Consequently, CMC is essentially a way of information transmission to some extent. Now, CMC has been applied to many fields, such as electronic commerce, online healthcare, distance learning, online communication, and online cooperation. In online healthcare, with the development of telemedicine and e-health, CMC through OHCs has become an important medium of patient–provider communication. CMC can negate geographical and temporal constraints, representing a convenient method by which physicians and patients can communicate.

Previous studies have explored physician–patient CMC from multiple perspectives, such as interaction frequency (3), interactional unfairness (27), communication competences (28), and interaction engagement (29). However, few studies have investigated the relationship between patients' self-disclosure and the establishment of patients' trust in physicians in the context of OHCs. Further, little research has sought to answer the following questions: what role does social support from physicians play in the relationship between patients' self-disclosure and the establishment of patients' trust in physicians? Does it act as a mediating variable affecting the relationship between physicians and patients? Additionally, few studies have explored the moderating effect of patients' hope-for-help motivation on the relationship between patients' self-disclosure and physicians' provision of social support.

### Patient Disclosure and the Establishment of Trust in Physicians

Self-disclosure is defined as the communication and presentation of personal information to another person (30–32), and plays an important role in the establishment of trust in others. Specifically, when individuals disclose personal information to one another, they improve understanding of each other and create trust (33). Social penetration theory (34) suggests that self-disclosure is a basic form of social exchange; as relationships develop, this exchange deepens and becomes more extensive. Meanwhile, Knapp's staircase model of relationships (35) also emphasised that self-disclosure can promote the intimacy of relationships and the formation of trust. The relationship between self-disclosure and trust is applicable to the physician–patient relationship. When a patient consults a physician, more self-disclosure on the part of the patient can promote interaction with the physician (36) and significantly improve the patient's experience, which leads to higher patient satisfaction (37). Additionally, when patients disclose more information to physicians, the physician–patient dialogue and contact increase, and the patients become more willing to trust the physicians (19, 20) and consult further with the physicians regarding their disease. Based on these findings, hypothesis H1 was proposed for the present study:

**H1:** Patients' self-disclosure positively influences the establishment of patients' trust in physicians.

### Mediating Effect of Physicians' Social Support

Generally speaking, social support can be divided into information support (38) and emotional support (39, 40). Information support involves providing actionable and objective information to a recipient. Meanwhile, emotional support, as a form of social support, involves empathising and providing emotional validation and encouragement (41). Regarding the relationship between self-disclosure and social support, Lee et al. (42) showed that individuals who disclose more information are more likely to receive social support. Meanwhile, Kim and Lee (43) suggested that honest self-disclosure positively affects the likelihood of receiving social support. Similarly, in the context of e-health, studies have found that the greater patients' self-disclosure through CMC, the more likely they are to receive physician feedback and social support (44). When patients communicate with physicians, disclosing more information indicates that they want the physicians to understand more about their symptoms and feelings. Furthermore, through high self-disclosure patients can obtain more information support (e.g., in regard to medication instructions and treatment plans) and emotional support (caring comments and tips) from physicians. Physicians can employ textual media to provide text-based social support to patients. However, they can also use richer media; for example, using voice media to provide voice-based social support to patients. Compared with text-based social support, voice-based social support can, through its greater richness, transmit more information (45, 46). Based on the above findings, we formulated hypotheses H2 and H3:

**H2:** Patients' self-disclosure positively influences physicians' text-based social support.
**H3:** Patients' self-disclosure positively influences physicians' voice-based social support.

More social support can increase patients' satisfaction with physicians' services (8), which can make them more willing to carry out further consultations with the physicians regarding their disease. Furthermore, higher level of social support also can often give rise to satisfactory social interaction and increase good psychological perception in physicians (47). Meanwhile, satisfactory social interaction can make patients perceive physicians as holding good intent towards themselves, and impel patients to consider that the information provided by physicians is more trustworthy (48). Besides, the social support can also increase people's health and well-being based on the social relationships, and it can transmit information, emotion, esteem among individuals (49). This can help patients improve the trust and reap more positive health outcomes (50, 51). Based on the above analysis, it can be seen that, not only does patients' self-disclosure have a direct effect on the establishment of trust in physicians, but it also has an indirect effect, through social support. Therefore, hypotheses H4 and H5 were formulated:

**H4:** Text-based social support from physicians mediates the relationship between patients' self-disclosure and the establishment of patients' trust in physicians.
**H5:** Voice-based social support from physicians mediates the relationship between patients' self-disclosure and the establishment of patients' trust in physicians.

### Moderating Effects of Patients' Motivation

Motivation represents an individual's psychological needs. Previous studies have shown that individuals' psychological needs have an important influence on the reception of social support during online disclosure (42, 52). Li et al. (53) suggested that individual psychological needs can impact the relationship between self-disclosure and social support. In other words, people with strong needs are more likely to disclose more information in order to receive more social support (53). On OHCs, when patients show hope-for-help motivation, this indicates that they are seeking social support. When patients express such motivation (e.g., through questions such as *Is my illness serious? Do I need surgery? How is it treated?*), physicians will find it easier to understand the patients' information demands. In such occasions, physicians will send more information to patients (26). According to the media-richness theory (45, 46), rich media can transmit more information than lean media, and rich media can also deliver multiple additional cues, such as voice tones, intonation, and emotions. During the online consultation process, if the patients present greater hope-for-help motivation, physicians will find it easier to understand the patients' information requests, and will consequently provide more social support. To enhance the effectiveness of their communication, physicians, upon noting hope-for-help motivation, may be more likely to use richer media (e.g., voice) to provide social support than leaner media (e.g., text). Besides, the rich media have faster and real-time feedback ability compared with the lean media. When the patients present hope-for-help motivation, sometimes there are urgent information in the motivation provided by the patients (e.g., through questions such as *Can I go to your hospital? What else needs to be checked? Do I need an operation as soon as possible? I am very anxious and fear for delaying my illness. I hope you can give me a suggestion*). At this time, to feed back information quickly, the physicians may choose a rich media (e.g., voice) to provide social support, other than a lean media (e.g., text). Therefore, hypotheses H6a and H6b were formulated.

**H6a:** Patient motivation weakens the effect of patients' self-disclosure on physicians' provision of text-based social support.
**H6b:** Patient motivation strengthens the effect of patients' self-disclosure on physicians' provision of voice-based social support.

**Figure 1:**
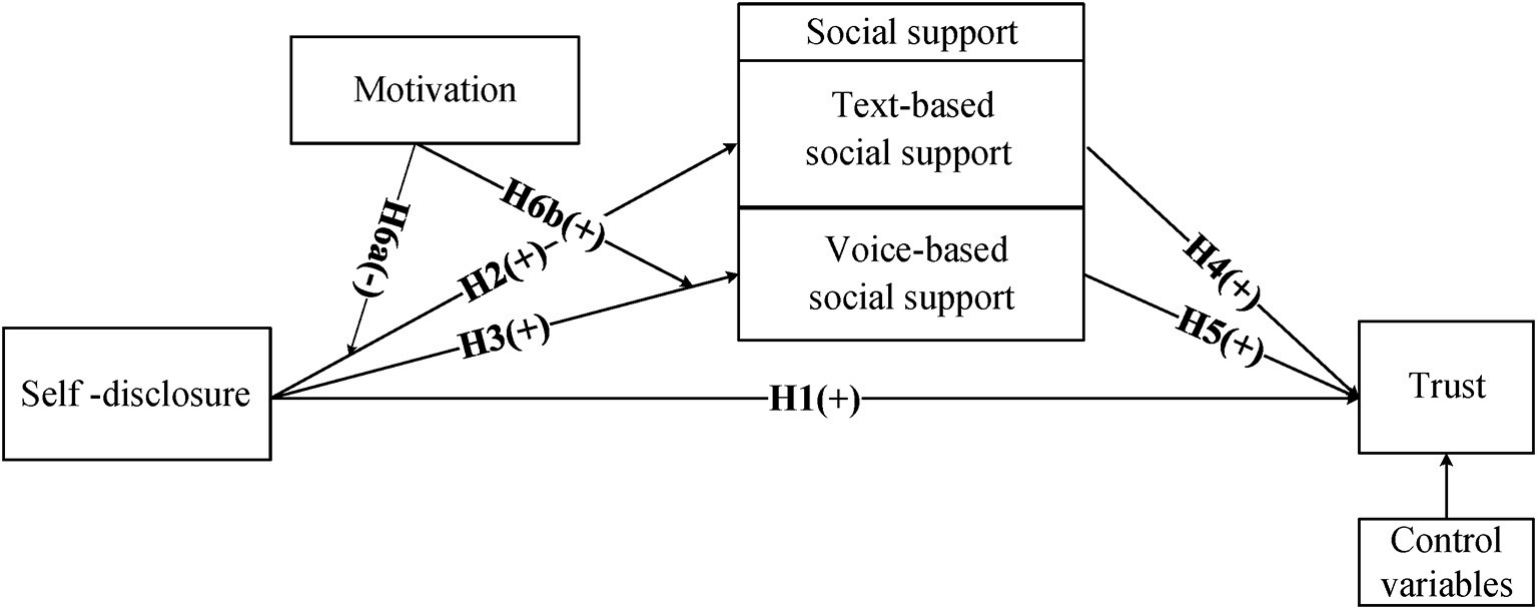
Research model.

The research model for the present study is shown in Figure 1.

## Materials and Methods

### Research Context

With the development of telemedicine and e-health, OHCs have become popular online medical consultation platforms. Good Doctor (haodf.com) is a China-based OHC that allows people to seek medical services for a fee. Good Doctor, which was created in 2006, is currently the largest e-health platform; at present, it features over 300 departments, covering over 3,000 diseases. The present research data are sourced from Good Doctor. We chose Good Doctor as the data source for two reasons. First, Good Doctor contains a great deal of data on many diseases, such as chronic diseases, serious diseases, and diseases that patients can be reluctant to disclose in public (high-privacy diseases). Second, Good Doctor contains data regarding physicians' attributes and patient–provider communication; such data greatly facilitated our research.

### Data Collection

**Figure 2:**
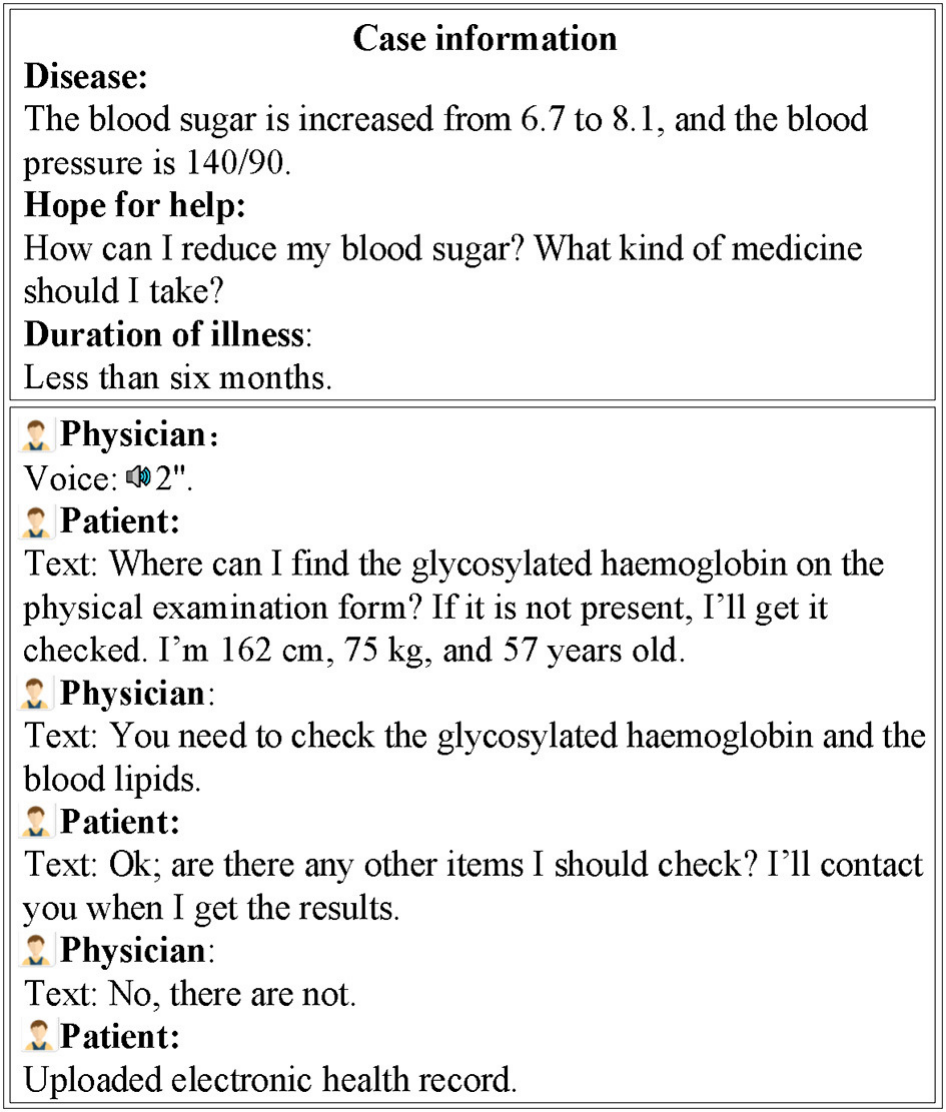
Example of a physician–patient communication record.

We collected from Good Doctor patient–provider communication data and data on physicians' attributes for November 2020. We used crawler software to download the data from the Good Doctor website. Thirty diseases were represented in our data, 10 high-privacy diseases and 20 common diseases. After downloading the data, we processed the data through the following steps: (1) deleted data that could not be recognised by a computer, (2) removed “space” characters, and (3) deleted data unrelated to our research (e.g., physicians' notes, tips). After applying these steps, the final dataset featured communication data from 1,537 physicians and 63,141 patients. The patient data contained 196,291 textual items, while the physician data contained 167,702 textual items and 41,538 voice items. The physicians' attribute data contained included physicians' clinical titles, educational titles, number of patients served, and total number of consultations. Thus, our final dataset featured physicians' attribute data and communication data. An example of doctor–patient communication on Good Doctor is shown in Figure 2.

### Operationalisation of Variables

The dependent variable was the establishment of patients' trust in the physicians. In e-business, sales of a product/service is an intuitive expression of consumers' trust in a business (54); similarly, in e-health, If the patients trust the physicians, the patients are likely to choose the physicians to consult the disease (20, 55, 56). For example, Gong (55) found that physicians' good service quality and reputation can enable patients to establish a trust relationship with them, and select these physicians in OHCs. Yoo et al. (56) considered that the trust propensity, platform reputation, and perceived physician credibility are important factors that can trigger patients' trust in physicians, and urge these patients to choose the physicians.Therefore, to some extent, the increment of patients who served by physicians can directly reflect the trust of patients to physicians. In our research, we used the increment of patients in the next month as a proxy variable to indicate the establishment of patients' trust in physicians. In this research, for each physician the increment in patients was obtained by subtracting the physician's number of patients in November 2020 from the number of patients in December 2020.

The independent variable was patients' self-disclosure; specifically, if the patients are willing to disclose more information, they will use more words to state their conditions. Contrarily, if they don't want to disclose information, they will employ less words or speak nothing to express their opinions and attitudes (57, 58). Therefore, the amount of disclosure is significantly correlated with the disclosure degree (58, 59). To effectively represent the amount and degree of patient disclosure, for each patient we used the average length (in characters) of their textual messages (PTL) to indicate the patient's level of self-disclosure. Physicians' social support was regarded as a mediating variable. Similarly, we used the average length (in characters) of the textual information (TL) and the duration (in seconds) of the voice information (VL) to quantify text-based and voice-based social support (57), respectively. Meanwhile, this study regards patients' hope-for-help motivation as a moderator variable, and this was quantified using the average length (in characters) of patients' hope for help messages. In addition, the number of textual messages patients send to physicians (PTN) may influence physician's provision of social support; concurrently, physicians who have a high clinical title (ClinicT), good reputation, and rich experience in medical diagnosis and treatment may provide more support to patients. Therefore, we controlled the influence of PTN, physicians' ClinicT, the time physicians spent online (OnlineT), the number of people who visited physicians' pages (VisitNum), the number of votes physicians received from patients (Votes, it is a kind of reputation which is the same as the gifts and thanks letter), and the price of consultation. The specific variable definitions are shown in Table 1.

**Table 1:** Variable description.

**Variable**	**Description**
**Dependent variable**	
Patients' trust in physicians (PT)	The increment in the number of patients consulting the physician over a 1-month period.
**Independent variable**	
Length of patient's textual messages (PTL)	The average length, in characters, of the textual information the patient sent to the physician.
**Mediating variables**	
Text-message length (TL)	The average length, in characters, of the textual information the physician sent to the patient.
Voice-message length (VL)	The average length, in seconds, of the voice information the physician sent to the patient.
**Control variables**	
Patient's number of textual messages (PTN)	The average number of textual messages that the patient sent to the physician.
Clinical title (ClinicT)	Clinical titles are awarded based on uniform national standards. For this study, titles were stratified into four stages (3 = medical director, 2 = associate medical director, 1 = chief physician, 0 = physician).
Time online (OnlineT)	The length of time the physician has been providing online consultations, from the first consultation to the time of data collection.
Number of visits (VisitNum)	The number of people who have visited a physician's page.
Votes	The number of votes physicians received from patients, it is a kind of reputation which is the same as the gifts and thanks letter.
Price	The price of an online written consultation with the physician.
**Moderator variable**	
Hope for help (HFH)	The average length, in characters, of hope-for-help messages sent by the patient.

The calculation formulas for *TL, VL, PTL*, and *PTN* described in Table 1 are presented below in formulae 1–4, respectively.


(1) $$TL=\frac{\sum_{\text{i=1}}^{\text{m}}\left(\sum_{\text{j=1}}^{{\text{n}}_{1}}\text{words}\_\text{number}\right)}{m}$$

(2) $$VL=\frac{\sum_{\text{i=1}}^{\text{m}}\left(\sum_{\text{j=1}}^{{\text{n}}_{2}}\text{voice}\_\text{time}\right)}{m}$$

(3) $$PTL=\frac{\sum_{\text{i=1}}^{\text{m}}\left(\sum_{\text{j=1}}^{{\text{n}}_{3}}\text{words}\_\text{number}\right)}{m}$$

(4) $$PTN=\frac{\sum_{\text{i=1}}^{\text{m}}{\text{n}}_{3}}{m}$$

In the above formulae, *TL* represents the average length (in characters) of the textual information physicians sent to patients, and *VL* represents the average length (in seconds) of the voice information physicians sent to patients. *PTL* represents the average length (in characters) of the textual information patients sent to physicians, and *PTN* represents the average number of textual messages patients sent to physicians. *m* indicates the number of patients the physician served during November 2020, *n*_1_and *n*_2_represent the number of text dialogues and voice dialogues, respectively, a physician had with a patient. *n*_3_ represents the number of text dialogues each patient had with each physician.

### Model Construction and Measurement

To test the above hypotheses, we applied a moderated mediation model approach, creating four models. To reduce the fluctuation of the data, we created a natural logarithm transformation for all variables; for convenience, the original variable name was used to express the processed variables. We used Model 1 to test the effect of patients' self-disclosure on the establishment of patients' trust in physicians.

**Model 1**

(5) $$\begin{array}{r} PT_{i}=\alpha_{1}+\beta_{1}PTL_{i}+\rho_{1}Control_{i}+\varepsilon_{i} \end{array}$$

Model 2 tests the effect of patients' self-disclosure on text-based and voice-based social support from physicians.

**Model 2**


(6) $$\begin{array}{r} TL_{i}=\alpha_{2}+\beta_{2}PTL_{i}+\lambda_{2}HFH_{i}+\rho_{2}Control_{i}+\varepsilon_{i} \end{array}$$

(7) $$\begin{array}{r} VL_{i}=\alpha_{3}+\beta_{3}PTL_{i}+\lambda_{3}HFH_{i}+\rho_{3}Control_{i}+\varepsilon_{i} \end{array}$$

Model 3 tests the mediating effect of physicians' provision of social support on the relationship between patients' self-disclosure and the establishment of patients' trust in physicians.

**Model 3**


(8) $$\begin{array}{r} PT_{i}=\alpha_{4}+\beta_{4}PTL_{i}+\gamma_{4}TL_{i}+\delta_{4}VL_{i}+\rho_{4}Control_{i}+\varepsilon_{i} \end{array}$$

Model 4 tests the moderating effect of patient motivation on the relationship between patients' self-disclosure and social support from physicians.

**Model 4**


(9) $$\begin{array}{l} TL_{i}=\alpha_{5}+\beta_{5}PTL_{i}+\lambda_{5}HFH_{i}+\theta_{5}HFH_{i}^{*}PTL_{i}+\rho_{5}Control_{i}\\ \;+\varepsilon_{i} \end{array}$$

(10) $$\begin{array}{l} \text{VL=}\alpha_{6}+\beta_{6}PTL_{i}+\lambda_{6}HFH_{i}+\theta_{6}HFH_{i}^{*}PTL_{i}+\rho_{6}Control_{i}\\ \;+\varepsilon_{i} \end{array}$$

In the above models, the specific meaning of parameters is shown in Table 2.

**Table 2:** Specific meaning of parameters in models.

**Parameters**	**Specific meaning**
*PT*_*i*_	The patients' trust in physician *i*.
*PTL*_*i*_	The length of the textual information that patient *i* sent to the physician.
*TL*_*i*_	The length of the textual information that physician *i* sent to the patient.
*VL*_*i*_	The length of the voice information that physician *i* sent to the patient.
*HFH*_*i*_	The text length of hope-for-help messages sent by patient *i*.
ε_*i*_	Error term.
α_1_, α_2_, α_3_, α_4_, α_5_, α_6_	Constant terms.
ρ_1_, ρ_2_, ρ_3_, ρ_4_, ρ_5_, ρ_6_	Coefficients of control variables.

## Results

The descriptive statistics of the variables are shown in Table 3, and the variable correlations are shown in Table 4. The mean variance inflation factor (VIF) was 2.71, and in all models the VIFs were <5. Therefore, there were no multicollinearity problems in our study. In this study, we use a moderation-mediation model to verify above hypotheses in our research. The moderation-mediation model can be divided into two parts: mediation model and moderation model. The mediation model contains model 1, model 2 and model 3. First of all, model 1 verifies the main effect between independent variable (PTL) and dependent variable (PT). If the coefficient β_1_ is significant, the main effect is effective. Secondly, the coefficient β_2_ and β_3_ must be significant in model 2. Finally, in model 3, the coefficient (γ_4_, δ_4_) of mediating variable (TL,VL) must be significant and the coefficient (β_4_) of independent variable (PTL) must be not significant. When above conditions are met, the mediating effect is effective. The model 4 is a moderation model and it can verify the moderation effect. In model 4, if the coefficient (β_5_, γ_5_, θ_5_ or β_6_, γ_6_, θ_6_) of independent variable (PTL), moderator variable (HFH) and the interactive item ( HFH^*^PTL) is significant, the moderating effect is effective. Table 5 presents the estimation results. Column (1) shows the estimation result for the control variables only. H1 predicted that patients' self-disclosure positively influences the establishment of patients' trust in physicians. Based on the regression result shown in column (2), β_1_ (β_1_ = 0.132, *t*= 3.19, ρ < 0.01) was positive and significant; thus, H1 was supported, and the main effect was verified.

**Table 3:** Descriptive statistics for the variables (*N* = 1,537).

**Variable**	**Mean**	**Standard deviation**	**Min**	**Max**
PT	71.15	149.6	0	4,716
PTL	78.51	75.27	0	1,538
TL	77.00	109.7	0	1,522
VL	11.57	28.31	0	411.7
PTN	2.624	2.801	0	75
ClinicT	2.407	0.737	0	3
OnlineT	6.943	3.336	0	12
VisitNum	3,120,000	7.153e + 06	2,616	148,000,000
Votes	318.4	341.6	2	3,142
Price	137.6	159.4	9	2,700
HFH	9.324	7.552	0	50

*The above variables are original values instead of the natural logarithmic transformations*.

*ClinicT: clinical title, HFH: hope for help messages, OnlineT: time online, PT: patients' trust in physicians, PTL: length of patient's textual messages, PTN: patient's number of textual messages, TL: text-message length, VisitNum: number of people who have visited a physician's page, VL: voice-message length*.

**Table 4:** Variable correlations.

**Variable**	**(1)**	**(2)**	**(3)**	**(4)**	**(5)**	**(6)**	**(7)**	**(8)**	**(9)**	**(10)**	**(11)**
1. PT	1										
2. PTL	0.273^***^	1									
3. TL	0.227^***^	0.741^***^	1								
4. VL	0.236^***^	0.324^***^	0.065^**^	1							
5. PTN	0.221^***^	0.883^***^	0.725^***^	0.272^***^	1						
6. ClinicT	0.100^***^	−0.034	−0.073^***^	−0.010	−0.086^***^	1					
7. OnlineT	0.065^**^	−0.003	−0.077^***^	0.017	−0.058^**^	0.369^***^	1				
8. VisitNum	0.401^***^	0.162^***^	0.096^***^	0.157^***^	0.109^***^	0.285^***^	0.634^***^	1			
9. Votes	0.544^***^	0.152^***^	0.072^***^	0.159^***^	0.074^***^	0.189^***^	0.404^***^	0.718^***^	1		
10. Price	0.217^***^	0.110^***^	0.060^**^	0.137^***^	0.056^**^	0.240^***^	0.222^***^	0.349^***^	0.394^***^	1	
11. HFH	0.073^***^	0.556^***^	0.572^***^	0.222^***^	0.545^***^	0.008	0.017	0.143^***^	0.096^***^	0.101^***^	1

* *p < 0.1*,

** *p < 0.05*,

*** *p < 0.01. The above variable values are natural logarithmic transformations*.

*ClinicT, clinical title; HFH, hope for help messages; OnlineT, time online; PT, patients' trust in physicians; PTL, length of patient's textual messages; PTN, patient's number of textual messages; TL, text-message length; VisitNum, number of people who have visited a physician's page; VL, voice-message length*.

**Table 5:** Estimated results.

**Column**	**(1)**	**(2)**	**(3)**	**(4)**	**(5)**	**(6)**	**(7)**
Dependent Variable	PT	PT	TL	VL	PT	TL	VL
PTL		0.132^***^	0.439^***^	0.347^***^	**0.050**	0.327^***^	0.420^***^
		**(3.19)**	**(8.52)**	**(7.58)**	**(1.11)**	(4.47)	(7.43)
TL					0.095^***^		
					**(3.21)**		
VL					0.092^***^		
					**(5.07)**		
PTN	0.319^***^	0.045	0.629^***^	−0.160	−0.017	0.819^***^	−0.283^*^
	(7.12)	(0.47)	(6.25)	(−1.21)	(−0.17)	(6.69)	(−1.96)
ClinicT	0.306^***^	0.292^***^	−0.108	−0.188	0.316^***^	−0.118	−0.182
	(2.87)	(2.75)	(−1.08)	(−1.24)	(2.99)	(−1.19)	(−1.20)
OnlineT	−0.576^***^	−0.579^***^	−0.215^***^	−0.212^**^	−0.538^***^	−0.215^***^	−0.212^**^
	(−8.87)	(−8.93)	(−3.17)	(−2.27)	(−8.50)	(−3.14)	(−2.27)
VisitNum	0.133^***^	0.134^***^	0.047^*^	0.107^**^	0.117^***^	0.0568^**^	0.101^**^
	(4.51)	(4.58)	(1.75)	(2.52)	(4.03)	(2.09)	(2.38)
Votes	0.593^***^	0.580^***^	−0.038	0.063	0.579^***^	−0.047	0.069
	(15.24)	(14.93)	(−1.07)	(1.13)	(14.96)	(−1.33)	(1.24)
Price	−0.012	−0.018	−0.007	0.123^***^	−0.030	0.008	0.113^**^
	(−0.40)	(−0.58)	(−0.23)	(2.72)	(−1.00)	(0.28)	(2.51)
HFH			0.328^***^	0.089^**^		0.283^***^	0.118^**^
			(8.25)	(2.25)		(7.71)	(2.54)
HFH*PTL						−0.080^***^	0.052^***^
						**(−3.27)**	**(2.65)**
Constant	−0.880^***^	−0.976^***^	0.696^***^	−1.903^***^	−0.861^***^	0.918^***^	−2.047^***^
	(−3.37)	(−3.76)	(2.69)	(−5.14)	(−3.33)	(3.29)	(−5.37)
Observations	1,537	1,537	1,537	1,537	1,537	1,537	1,537
R-squared	0.366	0.371	0.606	0.130	0.384	0.613	0.132
F	131.49	118.02	286.41	58.15	101.53	337.65	68.89
Prob > F	0	0	0	0	0	0	0

*t-statistics are in parentheses*;

* *p < 0.1*,

** *p < 0.05*,

*** *p < 0.01*.

*ClinicT, clinical title; HFH, hope for help messages; OnlineT, time online; PT, patients' trust in physicians; PTL, length of patient's textual messages; PTN, patient's number of textual messages; TL, text-message length; VisitNum, number of people who have visited a physician's page; VL, voice-message length*.

H2 and H3 predicted that patients' self-disclosure positively influences physicians' provision of text-based and voice-based, respectively, social support. Columns (3,4) indicate that β_2_ (β_2_ = 0.439, *t*= 8.52, ρ < 0.01) and β_3_ (β_3_ = 0.347, *t*= 7.58, ρ < 0.01) are positive and significant; thus, H2 and H3 are supported. This indicates that, during the process of patient–provider communication, an increasing amount of information is disclosed by patients, and physicians consequently obtain better understanding of patients' medical conditions and provide more effective treatment and social support to patients.

Column (5) tests the mediating effect of physicians' text-based and voice-based social support. In column (5), β_4_ (β_4_ = 0.050, *t*=1.11, ρ>0.1) is not significant, whereas γ_4_ (γ_4_ = 0.095, *t*= 3.21, ρ < 0.01) and δ_4_ (δ_4_ = 0.092, *t*= 5.07, ρ < 0.01) are positive and significant; this indicates that H4 and H5 were supported. These findings suggest that, in OHCs, if patients obtain more information and support from physicians, they can develop a more favourable perception of their medical experience and become more willing to build a trust relationship with the physicians. Thus, social support from physicians plays a complete mediating role in the relationship between patients' self-disclosure and the establishment of patients' trust in physicians.

**Figure 3:**
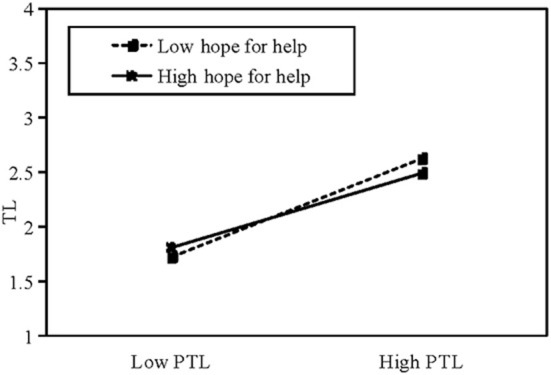
The mediating effect of hope-for-help motivation on TL. PTL: length of patient's textual messages.

**Figure 4:**
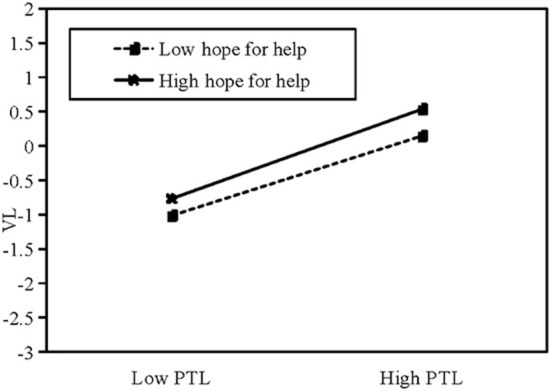
The mediating effect of hope-for-help motivation on VL. PTL: length of patient's textual messages.

Columns (6,7) test the moderating effect of patients' hope-for-help motivation on the relationship between patients' self-disclosure and physicians' social support. In columns (6,7), θ_5_ (θ_5_ = −0.080, *t* = −3.27, ρ < 0.01) is negative and significant, whereas θ_6_ (θ_6_ = 0.052, *t* = 2.65, ρ <0.01) is positive and significant; thus, H6a and H6b are supported. The specific moderating effect of hope-for-help motivation is shown in Figures 3, 4. This shows that patients' hope-for-help motivation weakens the effect of patients' self-disclosure on physicians' provision of text-based support, and strengthens the effect of patients' self-disclosure on physicians' provision of voice-based support.

### Robustness Cheque

To test the robustness of our results, we used data from December 2020 (communication data) and January 2021 (number of patients who consulted each physician) to verify our results. Table 6 shows that most of our results are robust and credible.

**Table 6:** Robustness cheque.

**Column**	**(1)**	**(2)**	**(3)**	**(4)**	**(5)**	**(6)**	**(7)**
Dependent Variable	PT	PT	TL	VL	PT	TL	VL
PTL		0.095^**^	0.398^***^	0.381^***^	**0.045**	0.189^***^	0.414^***^
		**(2.43)**	**(7.84)**	**(6.44)**	**(1.06)**	(2.74)	(5.78)
TL					0.061^**^		
					**(2.31)**		
VL					0.060^***^		
					**(4.00)**		
PTN	0.332^***^	0.137	0.650^***^	−0.286^*^	0.105	1.036^***^	−0.345^**^
	(7.57)	(1.57)	(6.37)	(−1.80)	(1.19)	(8.38)	(−1.98)
ClinicT	0.107	0.089	−0.253^**^	−0.238	0.115	−0.201^**^	−0.246
	(1.05)	(0.87)	(−2.39)	(−1.29)	(1.12)	(−1.98)	(−1.33)
OnlineT	−0.480^***^	−0.480^***^	−0.159^**^	−0.156	−0.462^***^	−0.160^***^	−0.155
	(−8.47)	(−8.46)	(−2.53)	(−1.46)	(−8.11)	(−2.65)	(−1.46)
VisitNum	0.105^***^	0.105^***^	0.051^*^	0.126^**^	0.093^***^	0.070^**^	0.123^**^
	(3.75)	(3.77)	(1.73)	(2.55)	(3.33)	(2.38)	(2.50)
Votes	0.539^***^	0.530^***^	−0.003	0.132^**^	0.523^***^	−0.043	0.138^**^
	(14.85)	(14.49)	(−0.08)	(2.10)	(14.48)	(−1.14)	(2.21)
Price	0.033	0.028	−0.040	0.056	0.027	0.010	0.047
	(1.20)	(1.01)	(−1.26)	(1.10)	(0.99)	(0.30)	(0.93)
HFH			0.247^***^	0.060		0.152^***^	0.075
			(6.04)	(1.18)		(3.99)	(1.31)
HFH*PTL						−0.169^***^	0.026
						**(−6.97)**	(0.94)
Constant	−0.324	−0.380	0.879^***^	−1.875^***^	−0.315	1.222^***^	−1.928^***^
	(−1.24)	(−1.45)	(3.14)	(−4.08)	(−1.20)	(4.28)	(−4.11)
Observations	1,428	1,428	1,428	1,428	1,428	1,428	1,428
R-squared	0.358	0.361	0.543	0.105	0.369	0.571	0.106
F	122.96	107.18	213.04	29.90	90.49	385.79	28.02
Prob > F	0	0	0	0	0	0	0

*t-statistics in parentheses*;

* *p < 0.1*,

** *p < 0.05*,

*** *p < 0.01*.

*ClinicT, clinical title; HFH, hope for help messages; OnlineT, time online; PT, patients' trust in physicians; PTL, length of patient's textual messages; PTN, patient's number of textual messages; TL, text-message length; VisitNum, number of people who have visited a physician's page; VL, voice-message length*.

## Discussion

Based on the Good Doctor medical platform, which is a big online health community in China, this study used a moderation-mediation model to explore the relationship among patients' self-disclosure, physicians' social support and the patients' establishment of trust in physicians. Additionally, it also tested the moderating effect of patients' motivation for “hope for help” on the physicians' social support. The results show that the patients' self-disclosure positively affect the patients' establishment of trust in physicians. The physicians' social support plays a complete mediating effect between the patients' self-disclosure positively and the patients' establishment of trust in physicians. Moreover, the patients' motivation for “hope for help” weakens the effect of patients' self-disclosure on physicians' provision of text-based social support and strengthens the effect of patients' self-disclosure on physicians' provision of voice-based social support. This research has some theoretical and practical contributions. First of all, this study is helpful to understand the patient-physician communication and patient-physician trust. It first establishes the contact between the patients' self-disclosure and the patients' establishment of trust in physicians from the patients. Secondly, this study extends the healthcare theory in OHCs and make the service and trust mechanism clear during the process of CMC. Thirdly, it provides the beneficial and essential practical implications for patients, physicians and the online health platforms. Overall, our research investigated the relationship between patients' self-disclosure, the physicians' social support and the establishment of patients' trust in physicians. It expands the health-care service delivery theory in e-health and has strong practical significance for patients, physicians, online medical platform, and the digital public health of human beings.

### Implications

This study provides insights for practise. First, our research provides a new approach for investigating the deep influencing mechanisms for the relationship between patients' self-disclosure and the establishment of patients' trust in physicians. Our research reveals a credible path by which patients can build trust in physicians during CMC. We found that patients' self-disclosure is helpful for building a harmonious and credible trust relationship between physicians and patients. Thus, when patients consult physicians, they should disclose as much information as possible.

Second, social support from physicians is significant for patients. Trust between physicians and patients is an essential element of physician–patient communication. If patients disclose more information to physicians, the physicians will provide more social support to the patients, and the patients will consequently become more willing to trust the physicians. The physicians should pay attention to the patients' self-disclosure, if there are more self-disclosure from patients, it means that the patients want to explain their symptoms and conditions clearly, the physicians should feed back the information as more as possible through text and voice media. At this time, the patients will consider that the physicians' service is worthy and they will trust physicians more. Meanwhile, if patients seek to obtain more social support from physicians, the patients could show more “hope-for-help” motivation to the physicians. Moreover, as a result of its richness as a medium, physicians should use voice media as much as possible when communicating with patients, as such media is helpful for providing increased social support and building a strong physician–patient relationship.

Third, CMC platforms could provide some modular options that help patients indicate their information needs. Such options could relate to medication, operations, diseases, and appointments. With such functionality, patients could disclose information more accurately and conveniently, and physicians could more easily understand patients' desires and, consequently, provide more social support.

### Limitations

There are some limitations to our study. First, the data in our research are sourced only from the Good Doctor website, which is a large online e-health community in China; future studies should use data from multiple online platforms to verify our hypotheses. Second, our research data are cross-sectional; future research should use panel data to obtain more robust results. Third, this paper focussed solely on information quantities to determine patients' levels of self-disclosure and physicians' levels of social support; future research should more closely consider the influence of communication content.

## Data Availability Statement

The original contributions presented in the study are included in the article/supplementary materials, further inquiries can be directed to the corresponding author.

## Author Contributions

All authors made substantial contributions to the research and agree with the content of the final manuscript.

## Funding

This work was supported by the National Natural Science Foundation Project (Grant No. 71871144), the Science and Technology Development Program of University of Shanghai for Science and Technology (Grant No. 2020KJFZ046), the Major Program of the National Fund of Philosophy and Social Science of China (Grant No. 18ZDA088), the Shanghai University of Finance and Economics Graduate Innovation Fund (Grant No. CXJJ-2019-400), and the Fund of Shanghai University of Sport (Grant No. A1-0303-21-0001-5).

## Conflict of Interest

The authors declare that the research was conducted in the absence of any commercial or financial relationships that could be construed as a potential conflict of interest.

## Publisher's Note

All claims expressed in this article are solely those of the authors and do not necessarily represent those of their affiliated organizations, or those of the publisher, the editors and the reviewers. Any product that may be evaluated in this article, or claim that may be made by its manufacturer, is not guaranteed or endorsed by the publisher.

## Abbreviations

ClinicT: Clinical title
CMC: Computer-mediated communication
HFH: Hope for help messages
OHC: Online health community
OnlineT: Time online
PT: Patients&#8217
trust in physicians
PTL: Length of patient's textual messages
PTN: Patient's number of textual messages
TL: Text-message length
VisitNum: Number of people who have visited a physician's page
VL: Voice-message length.
